# Synthesis and Characterization of Multiple Stimuli-Responsive Fluorescent Polymer Hydrogels Based on Terpyridine and *N*-Isopropylacrylamide

**DOI:** 10.3390/polym16111519

**Published:** 2024-05-28

**Authors:** Zihan Ma, Longhao Zhao, Chunhua Xie, Xianjian Wang, Ziyuan He, Xuegang Chen

**Affiliations:** Key Laboratory of Rubber-Plastic of Ministry of Education (QUST), School of Polymer Science and Engineering, Qingdao University of Science and Technology, Qingdao 266042, China; mazihan20212021@163.com (Z.M.); 18766206706@163.com (L.Z.); xch19993895815@163.com (C.X.); 15066470629@163.com (X.W.); hzy153090981778@126.com (Z.H.)

**Keywords:** fluorescent hydrogels, thermal response, pH response, terpyridine, *N*-isopropylacrylamide

## Abstract

A series of stimuli-responsive fluorescent hydrogels were successfully synthesized via micelle radical copolymerization of hydrophilic acrylamide (AM), hydrophobic chromophore terpyridine-based monomer (TPY), and *N*-isopropylacrylamide (NIPAM). These hydrogels presented blue emissions (423–440 nm) under room temperature, which is caused by the π-π* transition of the conjugated structures. Once the ambient temperature was increased to 55 °C, the fluorescence color changed from blue (430 nm) to pink (575 nm) within 10 min, subsequently to yellow (535 nm), and eventually back to pink. The thermal-responsive properties are attributed to the transition of the TPY units from unimer to dimer aggregation via the intermolecular charge transfer complex at high temperatures. The hydrogels showed pH-responsive properties. The emission peak of the hydrogel exhibited a blue shift of ~54 nm from neuter conditions to acidic conditions, while a 6 nm red shift to an alkaline environment was observed. The hydrogels demonstrated an obvious change in fluorescence intensity and wavelength upon adding different metal ions, which is caused by the coordination between the terpyridine units incorporated on the backbones and the metal ions. As a consequence, the hydrogels presented a sharp quenching fluorescence interaction with Fe^2+^, Fe^3+^, Cu^2+^, Hg^2+^, Ni^2+^, and Co^2+^, while it exhibited an enhanced fluorescence intensity interaction with Sn^2+^, Cd^2+^, and Zn^2+^. The microstructural, mechanical, and rheological properties of these luminescent hydrogels have been systematically investigated.

## 1. Introduction

A hydrogel is a hydrophilic polymer with three-dimensional network structures, which can absorb large amounts of water while retaining elasticity. Their elastic behaviors can be controlled through the modification of polymer chemical structures [1]. Fluorescent polymer hydrogels are novel soft materials that combine the properties of hydrogels and fluorescent polymers together. In most cases, the fluorescent function can be obtained by incorporating chromophores, such as organic fluorescent dyes, transition metal or rare earth complexes, conjugated moieties, quantum dots, and carbon dots into the hydrogel matrix with covalent coupling or physical interactions [2,3]. Polymer hydrogels with tunable fluorescent behaviors have received widespread attention. Some polymer hydrogels could present a tunable fluorescent behavior once they are under external stimuli such as temperature, ultrasound, pH value, chemical substances, ionic strength, light, etc. These polymer hydrogels are often promising since they can be applied in biomedical fields [4], chemical/biological sensors [5], optoelectronics, information display and encryption [6], and bionic actuators [7]. In recent decades, poly(*N*-isopropylacrylamide) (PNIPAM) has been widely adopted in the field of thermo-responsive hydrogels since it has a low critical solution temperature (LCST) of ~33 °C, in which the phase transition occurs in water. The hydrophilic and hydrophobic balance of the PNIPAM hydrogels can be changed in response to temperature variations, which facilitates thermally reversible phase separation for these hydrogels [8,9]. However, hydrogels with only a single performance response to environmental stimuli are insufficient for practical applications. Hydrogels that can change two or more properties under external stimuli will have a wider range of potential applications. In recent years, an increasing number of researchers have focused their attention on the development of fluorescent hydrogels capable of changing multiple properties under external stimuli. Chen et al. prepared a double-layer hydrogel actuator by combining the thermo-responsive GO-poly(*N*-isopropylacrylamide) hydrogel layer with a pH-responsive perylene bisimide-functionalized hyperbranched polyethylenimide hydrogel layer through supramolecular assembly, which exhibited complex shape deformation and a pH response switch for tunable fluorescence color [10]. Li et al. prepared a multi-stimuli-responsive mechanical/luminescent bi-anisotropic hydrogel by dispersing superparamagnetic nanoparticle-coated alumina (Fe/Al_2_O_3_) flakes in an aqueous solution with lanthanide luminescent centers, photochromic molecules, and *N*-isopropylacrylamide (NIPAM) and then aligning the alumina flakes with a magnetic field. The hydrogels realized a photo-reversible luminescence switch and thermo-reversible anisotropic deformation through the conformationally dependent fluorescence resonance energy transfer (FRET) between lanthanide donors and photochromic divinyl acceptors, as well as a phase transition of the polymerized NIPAM [11].

2,2′:6′,2″-terpyridine (terpy) exhibits strong coordination ability with some metal ions and the formation of stable complexes with well-defined structures. These complexes often present interesting photophysical properties involved in intramolecular/intermolecular charge transfer, metal–ligand charge transfer, photoinduced electron transfer, fluorescence resonance energy transfer, and so on [12]. Through the fine design of terpyridine-based molecules, ion-sensing smart hydrogel materials have been developed. Panda et al. reported double physical cross-linked luminescent hydrogels prepared via the copolymerization of acrylamide, methacrylic acid, and 6-(2,2′;6′,2″-terpyridine-4′-acyloxide)-hexyl acrylamide) [13]. By adjusting the molar ratio of terpyridine sensitizers and Eu^3+^/Tb^3+^, the hydrogels exhibited desirable mechanical properties, wide emission spectra, and self-healing properties. This is caused by the presence of a dynamic crosslinking interaction between the lanthanide sensitizer and the terpyridine. The hydrogel materials exhibited multiple stimuli-responsive capabilities such as pH value, temperature, metal ions, and mechanical strain. Harathi et al. synthesized novel water-soluble terpyridine-zinc(II) complex fluorescent probes, which exhibited fluorescence enhancement upon interaction with pyrophosphate (PPi) through photoinduced electron transfer (PET) inhibition. The probes were applied in the highly selective and sensitive detection of PPi ions in aqueous environments and living cells [14]. Wang et al. investigated the sensing behaviors of 2,2′:6′,2″-terpyridine-functionalized terthiophene (3T-Terpy) to metal ions, which revealed that the presence of Cd^2+^ resulted in a significant change in both the absorption spectrum and fluorescence emission maximum of the compound. This is attributed to the intramolecular charge transfer (ICT). Furthermore, a visual color change from green to yellow was observed under 365 nm UV light. 3T-Terpy demonstrated potential utility as a chemical sensor for Cd^2+^ through visualization and fluorescence detection [15]. The redox and photophysical characteristics of the terpyridine-based systems can be adjusted conveniently through modification with electron-withdrawing or electro-donating substituents [16]. Further research regarding the relationship between the chemical structures and the properties is urgent and plays a key role in the development of novel multiple stimuli-responsive fluorescent hydrogels and sensors.

**Scheme 1:**
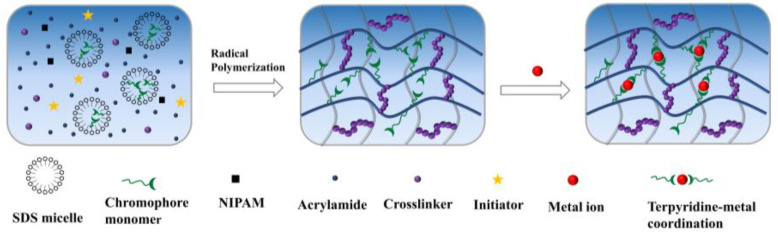
Schematic diagram of the preparation process of the fluorescent hydrogels by micellar radical copolymerization and metal ions’ response mechanisms.

Here, we reported a series of stimuli-responsive fluorescent hydrogels, which were obtained by the polymerization of the hydrophobic chromophore monomer 4′-(*N*-propenyl-4-pyridinio)-2,2′:6′,2″-terpyridine perchlorate (TPY), *N*-isopropylacrylamide (NIPAM) and hydrophilic monomer acrylamide (AM) via micellar radical copolymerization (Scheme 1). The packing form of the chromophores and the interaction of the hydrophobic groups in the polymer backbones might have a significant influence on the emission and mechanical properties. In addition, NIPAM units could endow the hydrogels with thermal-responsive performances due to their phase transformation ability at different temperatures.

Furthermore, coordination between the terpyridine in the polymer networks and the various metal ions imparts the hydrogels with ion-responsive behaviors, which can be used as sensors detecting metal ions. In this work, these relationships between the structures and functional properties were investigated in detail.

## 2. Materials and Methods

**Scheme 2:**
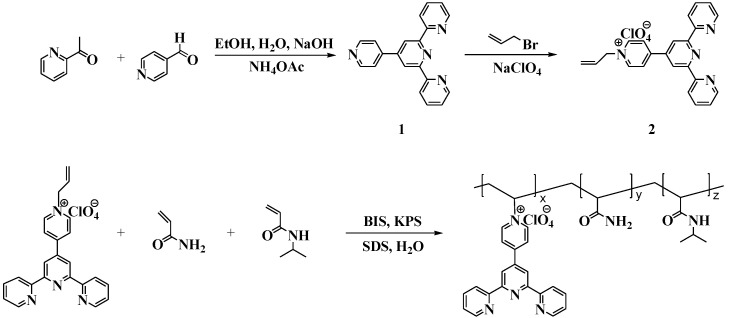
Synthesis route of the terpyridine functional monomer and the cross-linked polymer hydrogels.

The chemical reagents are commercially available and can be used directly without further purification. The synthesis of the monomer and the hydrogels with different compositions is described in Scheme 2. The ^1^H NMR spectra were determined using a Bruker Advance 500 MHz NMR spectrometer in DMSO-*d_6_* solution, with chemical shifts reported as ppm (TMS as internal standard). FT-IR was measured using a Bruker Vertex 70 Fourier transform spectrophotometer. Fluorescence measurement was performed using the F-4600 fluorescence spectrophotometer. The morphology of the hydrogels was determined using a scanning electron microscope (SEM, JEOL JSM-7500F). Before imaging, the samples were sputtered with Au. Rheological studies were performed on an ARES-G2 rheometer, and the thickness and diameter of the samples were 2 mm and 20 mm, respectively. The rheological measurements consisted of a frequency sweep and amplitude sweep at 25 °C. A frequency sweep was performed, which covered 0.1–100 rad/s at a strain of 1%, and an amplitude sweep was performed from 0.1 to 100% at a frequency of 6.28 rad/s to ensure that the obtained viscoelastic data are in the linear regime. Tensile strength was determined on the hydrogels using a universal testing machine (Instron 3343) at room temperature with a stretch rate of 50 mm/min. A sample was cut from the gel sheet with an initial standard length of 20 mm, a width of 10 mm, and a thickness of 1.2 mm. The nominal stress and strain were recorded, and Young’s modulus was calculated from the initial slope of the stress–strain curve with a strain below 10%. Compression strength tests were performed on the hydrogels with a compression rate set at 10 mm/min on a universal testing machine (UTM2502).

### 2.1. Synthesis of 4′-(4-pyridyl)-2,2′:6′,2″-terpyridine (**1**)

The mixture of 2-acetylpyridine (1.987 g, 16.4 mmol) and 4-pyridine carboxaldehyde (1.757 g, 16.4 mmol) was added to the methanol (30 mL) in a 100 mL three-neck flask. Then 12 mL of the NaOH aqueous solution (2.0 mol L^−1^) was added dropwise accompanied by stirring, followed by continuous stirring at 0 °C for 2 h. Then, 2-acetylpyridine (1.987 g, 16.4 mmol) and ammonium acetate (6.321 g, 82.0 mmol) were added to the reaction mixture. Subsequently, the mixture was refluxed at 80 °C for 5 h. The mixture was cooled to room temperature. The light-yellow solid was obtained by filtering. The crude product was washed with ethanol to produce light-yellow powder (yield 27.3%). ^1^H NMR (500 MHz, DMSO-*d_6_*): δ(ppm) 8.77 (t, J = 8.5 Hz, 6H), 8.69 (d, J = 8.0 Hz, 2H), 7.90 (t, J = 7.5 Hz, 2H), 7.80 (d, J = 5.5 Hz, 2H), and 7.38 (t, J = 5.75 Hz, 2H).

### 2.2. Synthesis of 4′-(N-propenyl-4-pyridinio)-2,2′: 6′,2″-terpyridine perchlorate (**2**)

A mixture of 4′-(4-pyridyl)-2,2′:6′,2″-terpyridine (1.77 g, 5.7 mmol) and 3-bromopropyl (3.45 g, 28.5 mmol) was added to acetonitrile (180 mL) under a nitrogen atmosphere. The mixed solution was stirred and refluxed for 24 h. After the mixture was cooled to room temperature, the precipitate was filtered and washed with acetonitrile. A white powder was obtained. Then, the white solid was dissolved in 200 mL of ethanol and excess NaClO_4_ was added. After filtration and drying, compound 2 (TPY) was obtained with a yield of 76.1%. ^1^H NMR (500 MHz, DMSO-*d_6_*): δ(ppm) 9.19 (d, J = 8.5 Hz, 2H),8.93 (s, 2H),8.81 (t, 4H),8.72 (d, J =10 Hz, 2H),8.09 (m, 2H),7.59 (m, 2H),6.23 (m, 1H),5.50 (m, 2H), and 5.37 (d, J =7.5 Hz, 2H).

### 2.3. Synthesis of the Fluorescent Hydrogels

TPY (94 mg, 0.2 mmol) was dissolved in 10 mL of a sodium dodecyl sulfate aqueous solution (70 mg/mL), which was stirred for 3 h to form a uniform emulsion. Subsequently, acrylamide (1.42 g, 0.02 mol), NIPAM (0.25 g, 2.2 mmol), methylene-bis-acrylamide (BIS) (0.12 g, 0.79 mmol, 3.5 mol% of total amount of the monomers), and potassium persulfate (KPS) (0.032 g, 0.12 mmol) was added to the solution, followed by stirring for another 1 h. The precursor solution was injected into a flat-bottom flask, which was kept at 60 °C for 3 h to complete the polymerization. The fluorescent hydrogel P-NIPAM_10_-TPY_0.9_ was obtained. Using a similar polymerization process, other hydrogels with different compositions were obtained (Table 1).

## 3. Results and Discussion

### 3.1. Synthesis and Characterization

The stimuli-responsive fluorescent hydrogels P-NIPAM_z_-TPY_x_ (Table 1) were prepared by one-pot micellar polymerization initiated by potassium persulfate (KPS). In the hydrogels, acrylamide was used as the main hydrophilic monomer, while TPY and *N*-isopropylacrylamide (NIPAM) were functionalized as the chromophore monomer and the thermosensitive monomer, respectively. The ratios of those three groups were different in the presence of the crosslinking agent *N*,*N*-methylene bisacrylamide (BIS) (Scheme 2). The micelle of sodium dodecyl sulfate (SDS) formed in the aqueous solution acts as a lipophilic environment capable of dissolving the hydrophobic TPY chromophobe monomer in the micelles [17]. This allowed for the successful copolymerization of the hydrophilic monomer with the hydrophobic monomer, resulting in the formation of chemically cross-linked hydrogels with stable structures. The hydrophobic units are distributed randomly along the polyacrylamide main chains.

**Figure 1:**
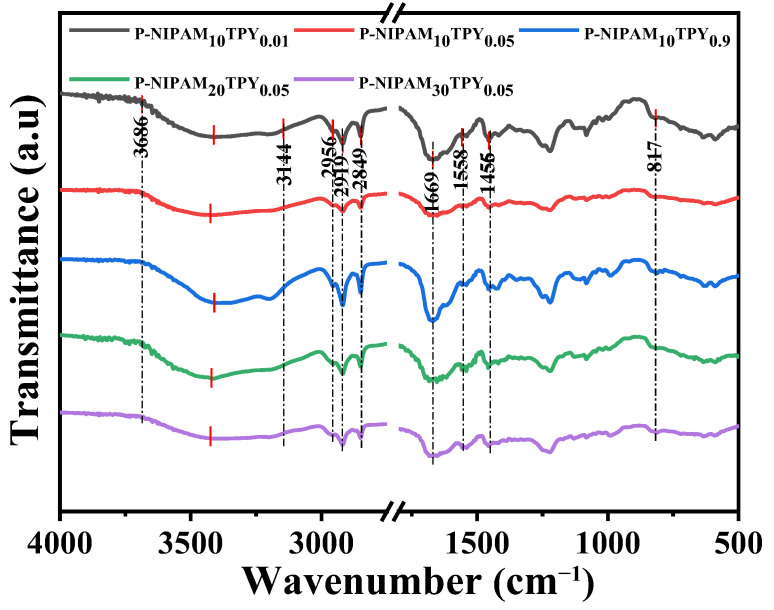
FT-IR spectra of the copolymers P-NIPAM_10_TPY_0.01_, P-NIPAM_10_TPY_0.05_, P-NIPAM_10_TPY_0.9_, P-NIPAM_20_TPY_0.05_, and P-NIPAM_30_TPY_0.05_.

The FTIR spectra of the polymers are shown in Figure 1. The wide peaks in the range of 3686–3144 cm^−1^ were attributed to the stretching vibrations of N-H, while the strong absorption peaks at ~1669 cm^−1^ were assigned to the stretching vibrations of C=O in the amide. The peaks at 2956 cm^−1^, 2919 cm^−1^, and 2849 cm^−1^ represent the typical stretching vibration of the saturated C-H in methyl. Methylene of the isopropyl groups belonged to NIPAM units [18], and their intensity increased with the increase in NIPAM unit content (Figure 1). Moreover, characteristic peaks of the aromatic ring skeleton (C=C) (~1456 cm^−1^), stretching vibration peaks of C=N in the TPY units (~1558 cm^−1^), and bending vibration of C-N in the pyridine heterocyclic ring (~817 cm^−1^) were also observed, which increased when enhancing the TPY content. There was no characteristic peak of vinyl C=C (approximately 1643 cm^−1^), which proved that the double bond on the monomer no longer existed. All of these indicate that the targeted copolymers were successfully synthesized [19].

### 3.2. Thermal-Responsive Properties

**Figure 2:**
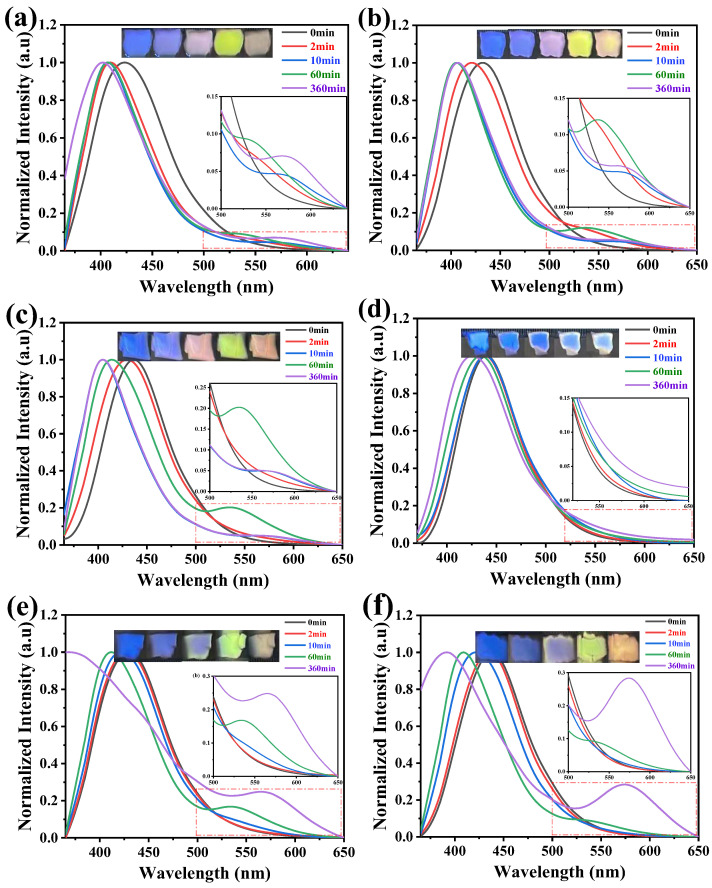
Fluorescence spectra of the polymers (**a**) P-NIPAM_10_TPY_0.01_, (**b**) P-NIPAM_10_TPY_0.03_, (**c**) P-NIPAM_10_TPY_0.05_, (**d**) P-NIPAM_10_TPY_0.9_, (**e**) P-NIPAM_20_TPY_0.05_, and (**f**) P-NIPAM_30_TPY_0.05_ under different heating times. Inset: Photos of the fluorescence of the hydrogels under different heating times.

Figure 2 shows the optical properties of these hydrogels, while the corresponding results are summarized in Table 2. At room temperature, the hydrogels P-NIPAM_10_TPY_0.01_, P-NIPAM_10_TPY_0.03_, P-NIPAM_10_TPY_0.05_, P-NIPAM_10_TPY_0.9_, P-NIPAM_20_TPY_0.05_, and P-NIPAM_30_TPY_0.05_ exhibited a blue fluorescence with emission peaks at 422, 431, 436, 440, 430, and 437 nm, respectively. These emission peaks were attributed to the π-π* transition of the conjugated moieties in the polymer backbones. Once the hydrogels were immersed in deionized water at 55 °C, the emission color of these hydrogels clearly changed. The fluorescent color of the hydrogels P-NIPAM_10_TPY_0.01_, P-NIPAM_10_TPY_0.03_, and P-NIPAM_10_TPY_0.05_ changed from blue to pink, then yellow, and finally back to pink (Figure 2a–c). The three hydrogels responded quickly within 10 min. The emission peaks of the hydrogels in the high-energy range exhibited slightly blue shift at approximately 13, 10, and 7 nm for P-NIPAM_10_TPY_0.01_, P-NIPAM_10_TPY_0.03_, and P-NIPAM_10_TPY_0.05_, respectively. There was a new broad emission peak that appeared in the low-energy range (near 570 nm) in these hydrogels, and consequently, the fluorescence color changed from blue to pink. After 60 min, the low-energy emission peak shifted from ~570 nm to ~530 nm, resulting in yellow fluorescence. After 6 h, the low-energy emission peak shifted from ~530 nm to ~570 nm again; meanwhile, the fluorescence color of the hydrogels returned to pink.

**Figure 3:**
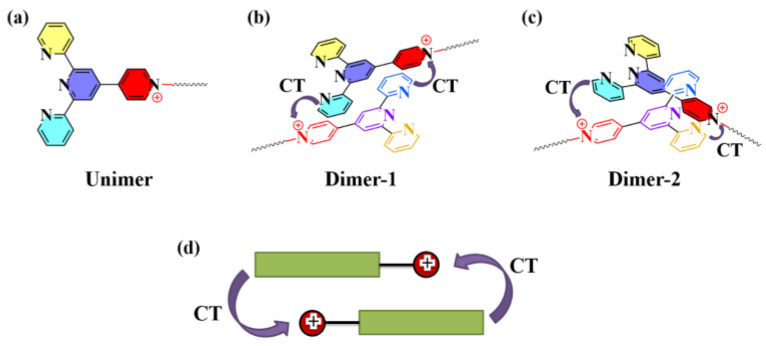
Schematic diagram of (**a**) unimer, (**b**) dimer-1, and (**c**) dimer-2 and (**d**) formation of the charge transfer (CT) complex.

The chromophore structure unit TPY was composed of the donor and acceptor properties together. The staggered stacking of the electron donors and acceptors of adjacent TPY units could form a dimer intermolecular charge transfer complex, resulting in the formation of a new intermolecular charge transfer (CT) band, which might generate additional low-energy emissions (Figure 3). The fluorescence color of the hydrogels changing from blue to pink and then to yellow was attributed to the coexistence of unimer and dimer (intermolecular CT complex) chromophores in the hydrogels, which resulted in blue and yellow emissions, respectively. There were two relatively stable forms of the dimer, namely, dimer-1 and dimer-2 (Figure 3).

In fact, the pink fluorescence of the hydrogels consisted of low-energy emissions of approximately 570 nm generated by dimer-1 and high-energy emissions of approximately 430 nm generated by the unimer, while the yellow fluorescence resulted from the combination of emissions at 535 nm generated by dimer-2 and emissions at approximately 430 nm generated by the unimer [20,21]. The tight stacking in dimer-2 enhanced electron separation, leading to a higher-energy gap compared to that of dimer-1; consequently, dimer-2 exhibited yellow emissions with a shorter wavelength (535 nm) compared to that of dimer-1 with pink emissions at a wavelength of 570 nm [21].

The fluorescence changes in the hydrogel P-NIPAM_10_TPY_0.9_ is shown in Figure 2d. Once the temperature was raised to 55 °C, there was no obvious change in the fluorescence color for the hydrogel P-NIPAM_10_TPY_0.9_, but the blue fluorescence color gradually became slightly lighter. The emission peak shifted from 440 nm to 425 nm after 360 min, and slight tailing in the long wavelength region appeared. It should be noted that no new characteristic peaks appeared, which implied that the CT complex dimer could not occur during the heating process. This was likely due to the disorder of the monomer aggregation caused by the excessive hydrophobicity of the chromophore TPY unit, which failed to form intermolecular charge transfer complexes. Consequently, in order to construct these thermal-responsive hydrogels with emission color changes, the appropriate content of the TPY unit should be 0.01–0.05%.

The hydrogels contain the thermal-responsive monomer NIPAM. Thus, the thermal-responsive properties were also observed through volume shrinkage. The hydrogel with 10% content of NIPAM presented ~1–5% volume shrinkage, while there was around 8–10% volume shrinkage regarding those with a content of NIPAM over 20%. Consequently, the hydrophilic–hydrophobic balance of the isopropylamide groups in NIPAM units changed with the increase in the ambient temperature. At lower temperatures, the hydrophilic segment (-CO-NH-) was likely to engage in hydrogen bond interactions with water molecules, leading to water absorption by the hydrogel network. With the increase in ambient temperature, the hydrogen bond associations were weakened or destroyed, while the hydrophobic interactions between hydrophobic groups (-CH(CH_3_)_2_) became stronger. This not only promoted the water molecules to escape from the networks but also the polymer chains to collapse, which might have resulted in the deformation and shrinkage of the hydrogels [8,22,23,24,25].

During the heating process, the volume of the hydrogels underwent shrinkage, which also caused the fluorescence color to change from yellow to pink. This might be due to the different responses in the structural stability of the two dimers of the chromophore TPY under the shrinkage of the hydrogels. dimer-1 exhibited better stability than dimer-2, which means dimer-1 forms a more compact conformation compared to dimer-2. During the shrinkage of the hydrogels, a transformation occurred regarding dimer-2 transforming into dimer-1, while the fluorescence color of the hydrogels changed from yellow to pink [21,24]. Pink fluorescence cannot be observed if the hydrogels contain 0.05% TYP and 20% or 30% NIPAM. This might be due to the fact that the thermal-responsive speed was too fast, which resulted in the fluorescence color changing directly from blue to yellow (Figure 2e,f). Obviously, the NIPAM units played a crucial role in the thermal-responsive speed of these hydrogels during the heating process.

### 3.3. Ion-Responsive Properties in Aqueous Solution

**Figure 4:**
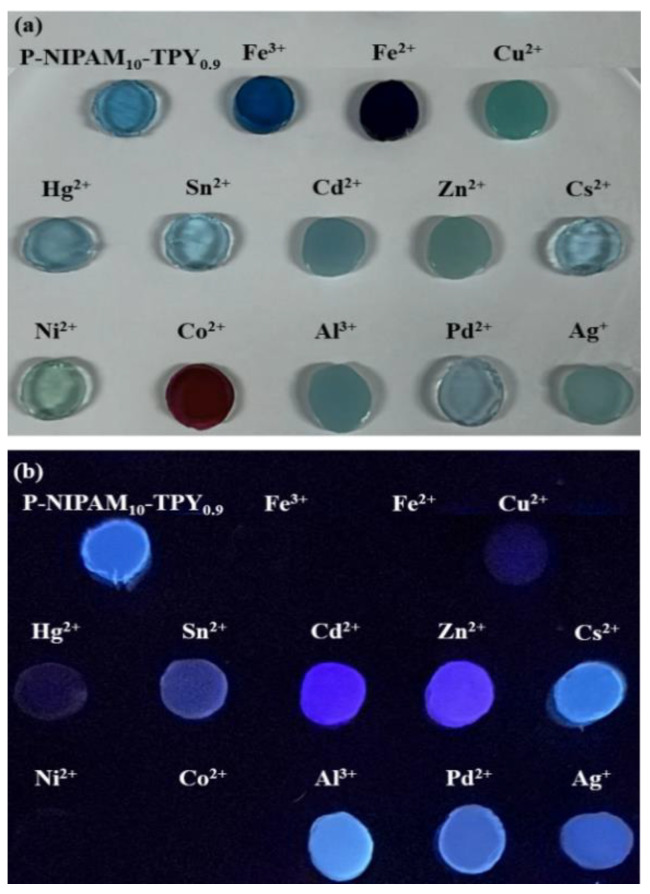
Photographs of the hydrogel P-NIPAM_10_TPY_0.9_ in aqueous solution of the different metal ions (1 × 10^−3^ mol/L) under visible light (**a**) and 365 nm UV light (**b**).

The ion-responsive properties of the hydrogel P-NIPAM_10_TPY_0.9_ were investigated using different metal ions (Fe^2+^, Fe^3+^, Cu^2+^, Hg^2+^, Sn^2+^, Cd^2+^, Zn^2+^, Cs^2+^, Ni^2+^, Co^2+^, Al^3+^, Pd^2+^, and Ag^+^) with a concentration of 1 × 10^−3^ mol/L in an aqueous solution (Figure 4). Upon contact with Fe^2+^, Fe^3+^, and Co^2+^, the color of the hydrogel P-NIPAM_10_TPY_0.9_ changed to black, dark blue, and deep red, while other metal ions changed to light green and light blue (Figure 4a). The color change was specific to each metal cation, allowing for its identification by the naked eye (dark blue for Fe^3+^, black for Fe^2+^, and deep red for Co^2+^), as illustrated in Figure 4a. The terpyridine moiety demonstrated a chelating ability toward transition metal cations, leading to the observed color change in the metal complexes. Therefore, the induction of the hydrogel relied on the monomer with a terpyridine moiety [26,27,28]. It is evident that the hydrogels displayed a wide range of ion-responsive properties and exhibited selectivity toward specific metal ions. The selectivity toward metal ions is further evidenced by the discernible colorimetric changes observed in the hydrogels, making them suitable for use as colorimetric sensors to selectively identify Fe^3+^, Fe^2+^, and Co^2+^.

**Figure 5:**
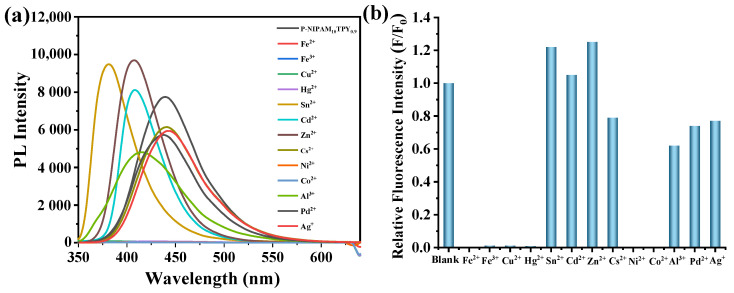
Fluorescence spectra (**a**) and relative fluorescence intensity of the hydrogel P-NIPAM_1_ PY_0.9_ (**b**) upon addition of different metal ions (1 × 10^−3^ mol/L).

Under excitation by 365 nm UV light, the fluorescence of the hydrogel was quenched by Fe^2+^, Fe^3+^, Cu^2+^, Hg^2+^, Ni^2+^, and Co^2+^ (Figure 4b). The influences of the different metal ions on the emission spectra of the hydrogel are depicted in Figure 5a. Adding Sn^2+^, Cd^2+^, and Zn^2+^ ions to the hydrogels could enhance their fluorescence, while causing a red shift in the fluorescence wavelength from 439 nm to 381, 408, and 407 nm, respectively. In contrast, adding Cs^2+^, Al^3+^, Pd^2+^, and Ag^+^ ions to the hydrogels could result in fluorescence quenching to a different extent, while Al^3+^ causes the hydrogel to undergo a distinct blue shift of 25 nm in the emission spectrum. The relative fluorescence intensity of the hydrogels’ interactions with different ions is shown in Figure 5b. As a versatile ligand, 2,2′:6′,2″-terpyridine exhibits a strong affinity for a variety of transition metal cations, which originates from both dπ-dπ* back bonds between the cations and the N heterocyclic rings, as well as the chelation effect. The interactions between the hydrogels and Sn^2+^, Cd^2+^, and Zn^2+^ led to fluorescence enhancement, which was caused by the coordination of terpyridine units with corresponding metal ions. There was a weak intramolecular charge transfer (ICT) in TPY units, and electron transfer occurred from three unmodified pyridine rings to the 4′-pyridine cores. After complexing with Sn^2+^, Cd^2+^, and Zn^2+^, the electron-donating characteristics of terpyridine units slightly decreased, which not only weakened the original intramolecular charge transfer process but also led to blue shifts in the fluorescence emissions to different degrees [20,29,30,31].

The fluorescence of the hydrogel was almost completely quenched (Figure 5) by the Fe^2+^, Fe^3+^, Cu^2+^, Hg^2+^, Ni^2+^, and Co^2+^ ions, which was in accordance with the phenomenon observed in Figure 4b. This might be due to the fact that the coordination of the metal ions with TPY units in the copolymer changes the configuration of electron clouds, which were also involved in metal–ligand charge transfer (MLCT) bands [32,33]. In addition, the coordination between metal ions and amide groups, as well as the corresponding electron transfer, was also likely responsible for the decrease in the fluorescence intensity [30,34]. Based on the wide ion-responsive properties, these hydrogels could be promising candidates for use as chemosensors to detect Fe^2+^, Fe^3+^, and Co^2+^ in aqueous solutions. Clearly, the detection of metal ions can also be accomplished by modifying the fluorescence emission properties, such as changes in intensity or wavelength shift, in response to different ions. As a result, these hydrogels show great promise as chemical sensors for detecting metal ions in aqueous solutions. Of course, the extensive coordination effect on metal ions also means this kind of hydrogel is expected to be applied to the adsorption of metal ions in water systems, especially for toxic and harmful ions.

### 3.4. pH-Responsive Properties

**Figure 6:**
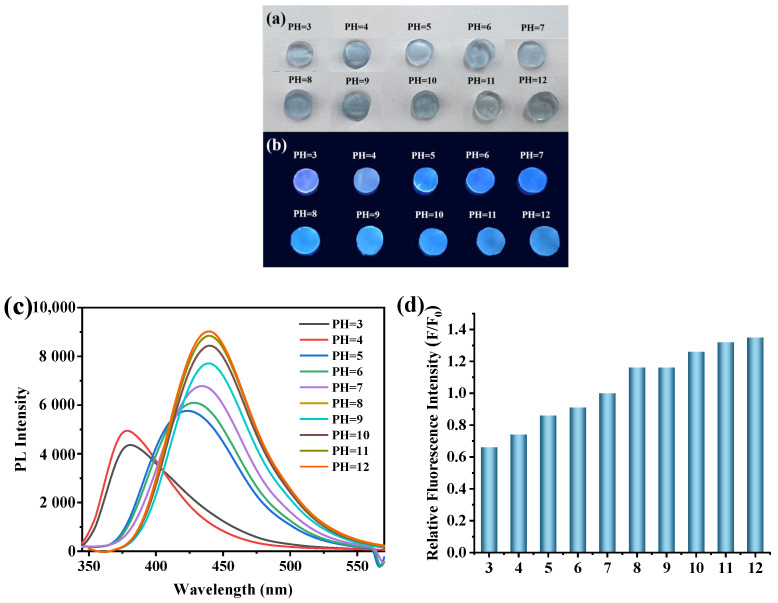
The photographs under visible light (**a**) and 365 nm UV light (**b**); the fluorescence spectra (**c**) and relative fluorescence intensity (**d**) of the hydrogel P-NIPAM_10_TPY_0.05_ under conditions of different pH values.

The hydrogels exhibited colorimetric changes under conditions with different pH values. As depicted in Figure 6a, the hydrogel P-NIPAM_10_TPY_0.05_ exhibited a light blue color when the pH was below 7, while the color became slightly darker when the pH value was above 7. The fluorescence appearance of the hydrogel in an aqueous solution with different pH values is shown in Figure 6b and the corresponding fluorescence spectra are illustrated in Figure 6c. When increasing the system’s acidity, the hydrogel always exhibited a blue color, but the fluorescence spectrum intensity gradually decreased. Meanwhile, the emission peak presented a blue shift with a decrease in pH value. The emission peak of the hydrogel exhibited a 56 nm blue shift from 434 nm to ~380 nm when the pH value decreased from 7 to 4 or 3. In addition, the fluorescence color of the hydrogel became light blue purple. This is ascribed to the gradual protonation of terpyridine moieties with a decrease in the pH value. This protonation not only enhanced the electron-withdrawing effect but also reduced the electron density around the protons. Therefore, the decrease in fluorescence intensity was likely attributed to the protonation of terpyridine, which reduced the intermolecular charge transfer effect [35,36]. The blue shift in the emission peak that appeared in an acidic environment might be attributed to two factors: (i) due to the coexistence of the unimer and dimer of TPY units in the copolymer, the decrease in the intermolecular charge transfer effect led to the disaggregation of the intermolecular CT complexes or (ii) the chromophore units preferred to exist in the form of the unimer [32,33,35,37]. With the increasing pH value, the emission peak of the hydrogel exhibited a small red shift with slightly increasing fluorescence intensity (Figure 6). When the pH value was 12, the emission peak of the hydrogel was 440 nm with a 6 nm red shift. This might be ascribed to the fact that the polymer backbones were stretched due to the deprotonation of terpyridine units in an alkaline environment [38,39].

### 3.5. Microstructures

**Figure 7:**
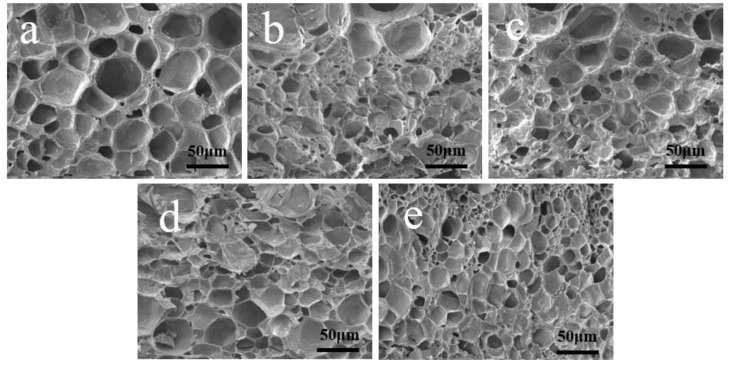
Scanning electron micrographs of the gels: (**a**) P-NIPAM_10_TPY_0.01_, (**b**) P-NIPAM_10_TPY_0.05_, (**c**) P-NIPAM_10_TPY_0.9_, (**d**) P-NIPAM_20_TPY_0.05_, and (**e**) P-NIPAM_30_TPY_0.05_.

To gain a deeper insight into the three-dimensional network structures within the hydrogels, scanning electron microscopy (SEM) was employed to examine the freeze-dried gel samples. Firstly, dry-gel P-NIPAM_10_TPY_0.01_ showed large and loose pore structures that were closed and complete, while the diameter of the gel pores was approximately 10–60 μm (Figure 7a). The pore diameter of dry-gel P-NIPAM_10_TPY_0.05_ (Figure 7b) was around 5–50 μm. Furthermore, the pores in dry-gel P-NIPAM_10_TPY_0.9_ (Figure 7c) exhibited a denser arrangement with apparent and uniform pore structures, of which the diameter was approximately 10–50 μm. When increasing the TPY content of the hydrogel, hydrophobic units aggregated with each other, which led to an increase in π-π stacking interactions. Thus, the pore structures of dry gels became dense and uniform, while the pore morphology was clear. Moreover, dry-gel P-NIPAM_20_TPY_0.05_ (Figure 7d) and P-NIPAM_30_TPY_0.05_ (Figure 7e) exhibited faveolate pore structures that were more compact and uniform, with pore diametersin the ranges of 10–40 μm and 5–30 μm, respectively. P-NIPAM_30_TPY_0.05_ has the characteristics of high porosity, good connectivity of pore structures, and uniform distribution of pore diameters. With the increase in NIPAM content, the number of hydrogen bonds that formed between hydrophilic amide groups and water molecules increased, which led to an improvement in the water absorption of the hydrogels. Once the water was discharged from the hydrogels after the freeze-drying process, porous structures of dry gels formed. Ice crystals that formed under freezing led to the formation of the faveolate hydrogels, while the pore networks could promote the diffusion of water molecules from the hydrogels. This process increased the density and regularity of the pore structures of dry gels, while small and dense three-dimensional networks were formed. The above results demonstrate that a high content of TPY and NIPAM functional units impedes macropore formation, thereby giving rise to a relatively uniform and diminutive pore structure in the range of several to tens of microns [8,40].

### 3.6. Rheological Behaviors

**Figure 8:**
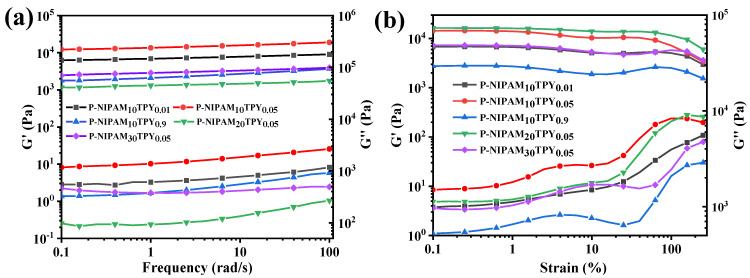
Variation in the storage modulus (G′) and loss modulus (G″) of the hydrogels P-NIPAM_10_TPY_0.01_, P-NIPAM_10_TPY_0.05_, P-NIPAM_10_TPY_0.9_, P-NIPAM_20_TPY_0.05_, and P-NIPAM_30_TPY_0.05_ as a function of the frequency (**a**) and strain (**b**) sweep at 25 °C.

Due to the three-dimensional network structures, the hydrogels exhibited characteristic viscoelastic behaviors. Rheology can be used to determine the energy stored by hydrogels and the energy dissipated in the system under oscillating stress with the storage modulus (G′) and loss modulus (G″), respectively. The rheological study (frequency and strain sweeps) of the hydrogels P-NIPAM_10_TPY_0.01_, P-NIPAM_10_TPY_0.05_, P-NIPAM_10_TPY_0.9_, P-NIPAM_20_TPY_0.05_, and P-NIPAM_30_TPY_0.05_ is shown in Figure 8. Throughout the frequency sweep from 100 to 0.1 rad/s, the value of G′ was much larger than G″. At the same time, both G′ and G″ increased with the enhancement of the sweep frequency. G′ and G″ of the hydrogels changed with the variations in the terpyridine content, which had a critical value of 0.05%. Within the range of 0.01%–0.05%, both G′ and G″ increased with the enhancement of the TPY content. Conversely, within the range of 0.05–0.9%, both G′ and G″ decreased as the TPY content increased. In the range of 0.01–0.05%, increasing TPY content might lead to the improvement of π-π stacking formation via the aggregation of hydrophobic units, which enhanced the hydrophobic interaction of the physical cross-linking. The combination of physical and chemical crosslinking resulted in dual cross-linking, which enhanced the storage modulus of the hydrogels. Once the content of terpyridine reached 0.9%, the high content of the hydrophobic terpyridine units hindered the formation of the uniform water-absorption networks in hydrogels, leading to a decrease in the storage modulus (G′). Furthermore, G′ and G″ varied with the change in NIPAM content, with 20% taken as a critical value. G′ and G″ of the P-NIPAM_10_TPY_0.05_ and P-NIPAM_30_TPY_0.05_ were higher than that of P-NIPAM_20_TPY_0.05_. G′ of the P-NIPAM_10_TPY_0.01_ and P-NIPAM_10_TPY_0.05_ was approximately 6273 Pa and 12,263 Pa, respectively, which indicated that the hydrogels presented good mechanical properties. The G″ of the P-NIPAM_30_TPY_0.05_ remained relatively constant, of which the value was high in the first stage and then decreased, while P-NIPAM_20_TPY_0.05_ consistently exhibited the lowest G″ value among these hydrogels.

The strain sweeps for the hydrogels from 1 to 1000% are demonstrated in Figure 8b. The G′ remained basically unchanged at the initial stage and subsequently fluctuated slightly, and then remained stable after recovery until yielding, at which time the hydrogels began to break with decreasing G′. The plateau storage moduli G′ for P-NIPAM_20_TPY_0.05_, P-NIPAM_10_TPY_0.05_, P-NIPAM_10_TPY_0.01_, P-NIPAM_30_TPY_0.05_, and P-NIPAM_10_TPY_0.9_ were approximately 16,690 Pa, 14,510 Pa, 7268 Pa, 6856 Pa, and 2816 Pa, respectively. G′ of P-NIPAM_20_TPY_0.05_ was the highest among these hydrogels, while G″ was relatively low. In addition, G″ increased sharply in the strain range of 25–160%. G′ and G″ of the P-NIPAM_10_TPY_0.9_ were the lowest among the five hydrogels. When the NIPAM content increased to 20%, the isopropyl groups in NIPAM aggregated as hydrophobic structures. This not only enhanced the entanglements of the polymer molecular chains in the hydrogels but also shortened the distance between polymer chains. As a consequence, the number of hydrogen bonds between molecular chains increased, which led to an increase in the value of G′. As the NIPAM content increased to 30%, the G′ value decreased, which indicated a decrease in elasticity and an increase in viscosity. The content of the hydrophilic units AM was relatively high when TPY and NIPAM content decreased. Meanwhile, tighter combinations between the AM units containing hydrophilic amide groups and water molecules formed through a strong hydrogen bonding association. Thus, the elasticity of the hydrogels increased and the hydrogels of P-NIPAM_10_TPY_0.05_ and P-NIPAM_10_TPY_0.01_ exhibited higher G′ values [22,41,42,43,44].

### 3.7. Mechanical Properties

**Figure 9:**
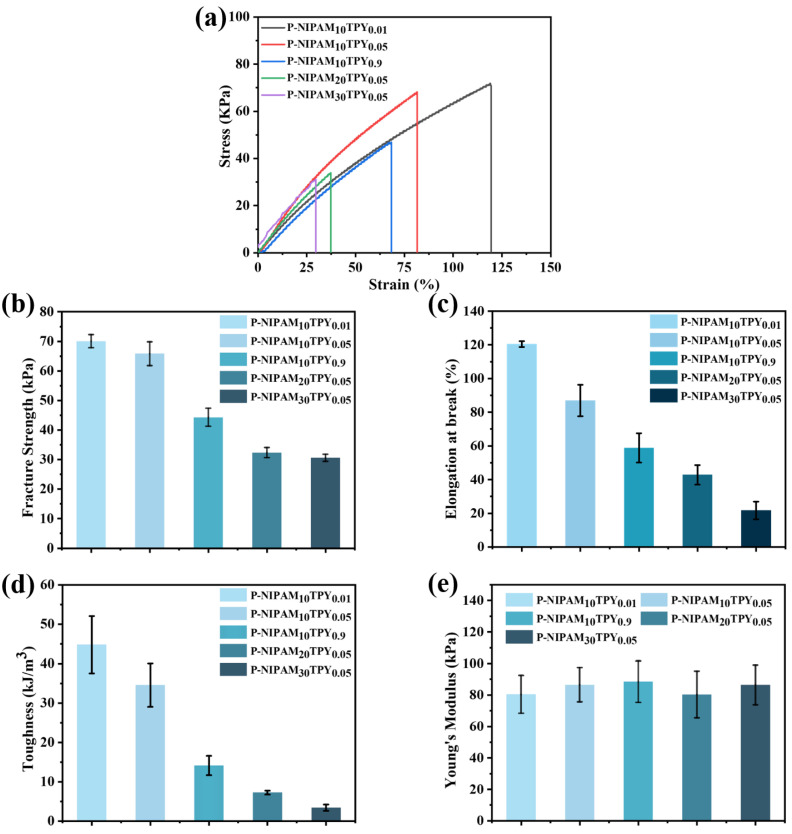
Tensile stress−strain curves (**a**), tensile breaking stress σ_b_ (**b**), breaking strain ε_b_ (**c**), toughness (**d**), and Young’s modulus E (**e**) of the hydrogels with different components.

The tensile stress–strain curves and the detailed results of the hydrogels are shown in Figure 9. As the TPY content decreased, the tensile breaking strength δ_b_, breaking strain ε_b_, and toughness of the hydrogels gradually increased, reaching 71 kPa, 120%, and 45 kJ/m^3^ for the P-NIPAM_10_TPY_0.01_ hydrogel with a TPY content of 0.01%. There was an upward trend for Young’s modulus E with an increase in the TPY content, which reached 88 kPa for the P-NIPAM_10_TPY_0.9_ hydrogel with a TPY content of 0.9% (Figure 9e). Moreover, the δ_b_, ε_b_, and toughness of the hydrogels decreased as the NIPAM content increased. Young’s modulus E of P-NIPAM_10_TPY_0.05_ and P-NIPAM_30_TPY_0.05_ exhibited relatively consistent values, while the E value of P-NIPAM_20_TPY_0.05_ experienced a slight decrease. The tensile measurement results demonstrated that the hydrogels with low TPY and NIPAM contents had favorable values of δ_b_, ε_b_, and toughness while increasing the TPY and NIPAM content led to an increase in E from 80 kPa to 88 kPa. The increase in TPY content enhanced the π–π stacking interaction formation through mutual aggregation of the hydrophobic units. The expansion of the π system by the 4′-pyridine groups in the TPY units resulted in the interaction of large conjugated π structures as well as rigid polypyridine groups in the terpyridine ligands. Consequently, the mechanical properties of the hydrogels were modified, such as increasing Young’s modulus E and brittleness and decreasing the toughness. On the other hand, because the isopropyl groups in the NIPAM units were hydrophobic groups, the increase in the NIPAM content likely not only interfered with the hydrophilic–hydrophobic balance of the molecular chains but also caused polymer entanglement. This might enhance the number of formations of hydrogen bonds between water molecules and -CO-NH-. Thus, more energy was needed to destroy the hydrogen bonds when subjected to an external force; that is, the E value of hydrogels was enhanced [45,46].

**Figure 10:**
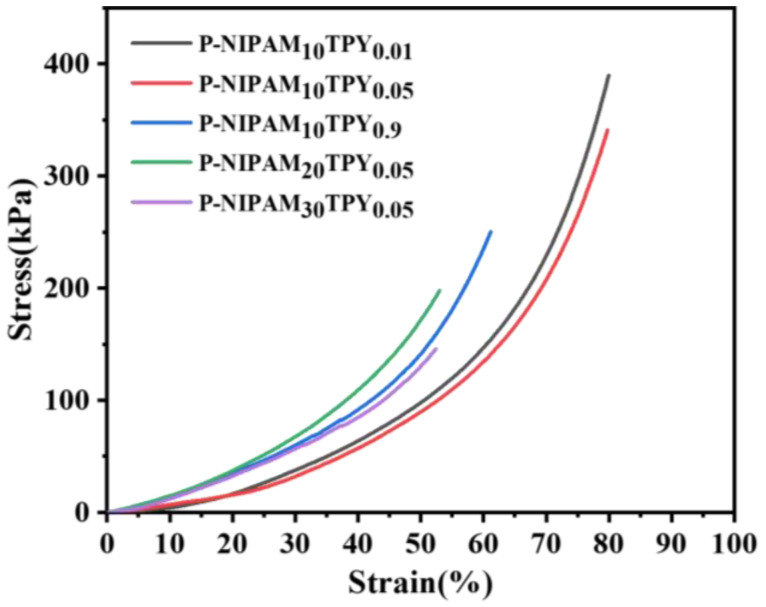
Compression stress–strain curves of the hydrogels with different components.

The compressive strength of the hydrogels was evaluated, and the compressive stress–strain curves are shown in Figure 10. At the compression rate of 10 mm/min, the compressive strain of P-NIPAM_10_TPY_0.01_ and P-NIPAM_10_TPY_0.05_ could reach 80%, while the corresponding compressive stresses were 390 kPa and 340 kPa, respectively. When the strain was at an intermediate value of 40%, the compressive stresses of the P-NIPAM_10_TPY_0.01_, P-NIPAM_10_TPY_0.05_, P-NIPAM_10_TPY_0.9_, P-NIPAM_20_TPY_0.05_, and P-NIPAM_30_TPY_0.05_ hydrogels was 64 kPa, 58 kPa, 93 kPa, 112 kPa, and 86 kPa, respectively. After the pressure was removed, the hydrogels could completely recover, which indicated that the hydrogels had good elasticity. Obviously, the lower content of TPY and NIPAM endowed the resulting hydrogels with good elasticity and larger compressive strength, while the rigidity and the compressive at low compressive strain were not higher than that of the hydrogels with a high content of TPY and NIPAM units. This was due to the fact that after increasing the content of TPY and NIPAM from the hydrogel systems, more physical cross-linking was formed based on π–π interactions from TPY and hydrophobic interactions from NIPAM moieties [47].

## 4. Conclusions

In summary, stimuli-responsive fluorescent hydrogels were successfully prepared through micellar radical copolymerization of the hydrophilic acrylamide with the thermosensitive monomer NIPAM and the hydrophobic chromophore monomer TPY. The hydrogels exhibited thermal-responsive properties with quick fluorescence changes and volume shrinkage to different extents. Under different environmental conditions, the TPY units in the hydrogels can display various forms such as unimer and dimer (intermolecular charge transfer complex), which produce blue and yellow emissions, respectively. Upon increasing the temperature, the TPY units can be transitioned from unimer to dimer-1 and dimer-2, which leads to sequential color changes from blue to pink and yellow, and eventually back to pink. Furthermore, the hydrogels also exhibited sensing properties to metal ions and pH values with fluorescence color changes. Based on the effect of terpyridine–metal coordination on the charge transfer and electron distribution, the hydrogels were highly responsive to various metal ions. The fluorescence of the hydrogels was significantly quenched by Fe^2+^, Fe^3+^, Cu^2+^, Hg^2+^, Ni^2+^, and Co^2+^, while the fluorescence intensity was enhanced by Sn^2+^, Cd^2+^, and Zn^2+^ to different degrees. The response of the hydrogels to the environmental pH value was in the range of 3–12, which was influenced by protonation and deprotonation of the terpyridine units. There was an approximate 54 nm blue shift in the fluorescence peak in acidic conditions and an approximate 6 nm red shift in an alkaline environment. Additionally, the changes in TPY and NIPAM content had significant influences on the micromorphology, rheology, and mechanical properties of the hydrogels. If the TPY content was between 0.01 and 0.05% and the NIPAM content was 10%, small and dense faveolate three-dimensional network pore structures of the hydrogels, along with good elastic modulus and mechanical properties, were formed. Overall, this study serves as a reference for advancing the research and application of stimuli-responsive fluorescent hydrogels in environmental responses, ion detection, and chemical sensing.

## Figures and Tables

**Table 1:** Hydrogels with different components.

Hydrogel	NIPAM (mol %)	TPY (mol %)	AM (mol %)
P-NIPAM_10_-TPY_0.01_	10.00	0.01	89.99
P-NIPAM_10_-TPY_0.03_	10.00	0.03	89.97
P-NIPAM_10_-TPY_0.05_	10.00	0.05	89.95
P-NIPAM_10_-TPY_0.9_	10.00	0.90	89.10
P-NIPAM_20_-TPY_0.05_	20.00	0.05	79.95
P-NIPAM_30_-TPY_0.05_	30.00	0.05	69.95

**Table 2:** Emission peaks (λ_max_) of the hydrogels with different compositions with different heating times.

Hydrogels	λ_max_/nm				
	0 min	2 min	10 min	60 min	360 min
P-NIPAM_10_-TPY_0.01_	422	409	406,572	407,530	402,570
P-NIPAM_10_-TPY_0.03_	431	421	406,573	405,534	406,569
P-NIPAM_10_-TPY_0.05_	436	429	404,574	413,535	404,572
P-NIPAM_10_-TPY_0.9_	440	438	439	434	425
P-NIPAM_20_-TPY_0.05_	430	427	421	411,534	368,567
P-NIPAM_30_-TPY_0.05_	437	435	421	408,529	391,574

## Data Availability

The data are contained within the article.
